# The Efficacy of Pembrolizumab Immunotherapy in the Treatment of Endometrial Cancer: A Systematic Review

**DOI:** 10.3390/ijms26188789

**Published:** 2025-09-09

**Authors:** Natalia Picheta, Julia Piekarz, Krzysztof Kułak, Rafał Tarkowski

**Affiliations:** 1 Student’s Scientific Association at the I Chair and Department of Gynaecological Oncology and Gynaecology, Medical University of Lublin, Staszica St. 16, 20-081 Lublin, Poland; natalia.picheta2812@gmail.com (N.P.); piekarzjulia1@gmail.com (J.P.); 2 I Chair and Department of Gynaecological Oncology and Gynaecology, Medical University of Lublin, Staszica St. 16, 20-081 Lublin, Poland; rafaltar@yahoo.com

**Keywords:** pembrolizumab in endometrial cancer, immunotherapy in endometrial cancer, pMMR pembrolizumab, molecular-type endometrial cancer immunotherapy, pembrolizumab vs. chemotherapy in gynecological cancer

## Abstract

Endometrial cancer represents one of the most common gynecological cancers in women. In recent years, there has been increasing interest in immunotherapy, including the use of pembrolizumab, particularly for the treatment of cancers with deficient mismatch repair (dMMR) and microsatellite instability-high (MSI-H). A systematic review of the literature from 2020 to 2025 was conducted according to the PICO model. Six studies were included in this review, comprising four randomized clinical trials (RCTs) and two pre-specified subgroup analyses derived from previous RCTs involving a total of 3684 patients with early-stage or advanced disease or metastatic or recurrent endometrial cancer. Interventions included the use of pembrolizumab in monotherapy and in combination with chemotherapy or lenvatinib. Pembrolizumab showed a significant improvement in progression-free survival (PFS) and overall survival (OS) in the dMMR patient groups. Therapeutic benefit was limited in the proficient mismatch repair (pMMR) groups. The incidence of side effects was high but comparable to the control group. Pembrolizumab, especially in combination therapy with lenvatinib, is a promising therapeutic option for patients with dMMR/MSI-H endometrial cancer. The results suggest a potential long-term treatment effect, although the limitations of the RCT and the variability in the therapeutic regimens require further research.

## 1. Introduction

Endometrial cancer is the most common malignant tumor of the reproductive system and the fourth most common malignant tumor diagnosed in women in developed countries [1]. By 2040, endometrial cancer is projected to become the third most commonly diagnosed and the fourth leading cause of cancer-related death among women [2]. According to WHO data from 2020, 417,367 new cases were diagnosed worldwide, while 97,370 deaths were recorded. In 2019, the age-standardized incidence rate (ASIR) in Western Europe was 27.8 per 100,000 women, and in Eastern Europe, it was 27.5. The age-standardized mortality rate (ASMR) was 3.7 and 3.2 per 100,000 women, respectively [3]. Forecasts indicate a further upward trend in morbidity and mortality in the coming decade [4]. The peak incidence is observed in patients aged 65 to 75 years. Risk factors include obesity, estrogen replacement therapy, tamoxifen therapy, early menarche, late menopause, and genetic abnormalities associated with Lynch syndrome [5]. In over 90% of patients, the disease manifests with abnormal uterine bleeding and pelvic pain.

Diagnosis is based on transvaginal ultrasound examination with sampling and histological examination [6]. The prognosis of patients depends primarily on the stage of disease determined by the International Federation of Gynecology and Obstetrics (FIGO) [7]. Stage I is characterized by a 5-year survival rate of 85%, while that in stage IV is up to 25% [8]. The basis of cancer therapy is radical surgery. In the early stages, hysterectomy with bilateral salpingo-oophorectomy is performed, while in advanced cases, cytoreduction is performed if technically possible [9]. In patients with advanced cancer and relapsed disease, systemic treatment is used. With standard first-line treatment based on carboplatin and paclitaxel, the overall response rate (ORR) was 40–50%, while the median PFS and OS were 14 and 32 months, respectively [10]. In addition, hormone therapy is used as monotherapy or in combination with, for example, mTOR inhibitors. One study showed that the combination of letrozole with everolimus showed a Clinical Benefit Rate (CBR) of 40% [11]. Despite the available treatment options, disease recurrence is observed in a significant proportion of patients. In the early stage, recurrence occurs in 10–15% of patients and in the advanced stage in 40–70% of patients, which significantly reduces patients’ quality of life and results in a decrease in overall survival (OS) [12]. Immunotherapy appears to be the remedy for prolonging OS in patients with recurrent and advanced endometrial cancer. Studies show a clear prolongation of OS and progression-free survival (PFS) [13].

## 2. Methods

### 2.1. Review Design and Search Strategy

This study focuses on comparing these pembrolizumab therapies with standard chemotherapy. The PICO model was used to organize and guide the literature review.

- Population: The target population is patients over 18 years of age with advanced, metastatic, or recurrent endometrial cancer, regardless of histological type, excluding sarcomas and angiosarcomas.
- Intervention: Interventions include the use of pembrolizumab therapy, either in monotherapy or together with chemotherapy or lenvatinib.
- Comparison: The efficacy and safety of pembrolizumab therapy for the treatment of endometrial cancer were compared with those of standard cancer treatments. This comparison will allow for an assessment of the relative risks and benefits of the said therapy.
- Results: The results analyzed include the efficacy and safety of pembrolizumab therapy for the treatment of endometrial cancer. Efficacy is measured by PFS and OS. In addition, the frequency of side effects events associated with the use of pembrolizumab will be assessed.

The search strategy used the most important inclusion criterion: the selection of primary studies of the RCT type. The focus was on these types of studies because they offer the least risk of bias and provide high reliability of scientific evidence. Case studies, animal studies, and other studies were omitted. A criterion that was as important for the inclusion of studies was the time of publication of these studies (last 5 years).

Database search terms: PubMed, ClinicalKey, and SpringerLink covered the years 2020–2025 to ensure the most up-to-date data. Keywords used: “pembrolizumab in endometrial cancer”: ‘immunotherapy in endometrial cancer’; “pMMR pembrolizumab”; ‘molecular type endometrial cancer immunotherapy’; ‘pembrolizumab vs. chemotherapy in gynecological cancer’. A manual search of retrieved studies was performed to ensure that RCTs were not missed. Two authors independently examined the databases and carefully assessed the articles. Inconsistent issues were resolved through discussion with two other authors.

### 2.2. Data Collection

This review included six studies involving 3684 patients in which pembrolizumab with either chemotherapy or lenvatinib was administered in the intervention group. The RCTs provided sufficient data on the PFS, OS, and status of patients receiving the specified treatment, including the side effects of therapy.

### 2.3. Assessment of Risk of Bias in Included Studies

All six randomized trials included in the analysis were subjected to a risk of bias assessment using the updated Cochrane RoB 2.0 tool. The assessment covered five key areas: (1) randomization procedure, (2) compliance of the intervention with the protocol design, (3) completeness of outcome data, (4) reliability of outcome measurement, and (5) selectivity of outcome reporting.

Two independent reviewers analyzed each study against the criteria listed above, assigning a risk level as low, high, or uncertain (“some concerns”). In the event of divergent assessments, a decision was made jointly through discussion. If necessary, a third author mediated the decisions, acting as an arbitrator.

## 3. Results

### 3.1. Selection and Identification of Studies

**Figure 1:**
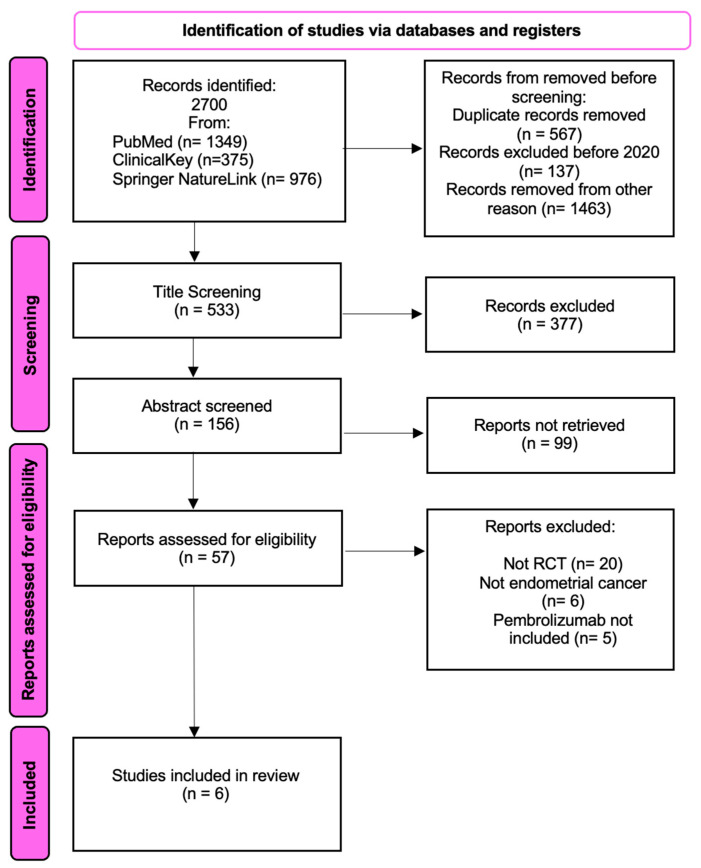
Preferred Reporting Items for Systematic Reviews and Meta-analyses (PRISMA) flow diagram of study identification, inclusion, and exclusion.

A systematic search of the databases yielded 2700 records. A total of 567 duplicate records were removed. Additionally, 137 records that did not meet the time criteria and 1463 records that did not meet the criteria were excluded due to a lack of significance of the results or effects. Further records were excluded because they were not RCTs, did not include endometrial cancer, and did not include pembrolizumab in the intervention. Finally, six articles were included in the main analysis. After assessing the quality of the included studies, six studies were classified as good-quality, involving 3684 patients. The protocol for this systematic review was preregistered (private at the time of submission) on the Open Science Framework (OSF) database (osf.io/ak3jr). The detailed data selection and identification process is shown in Figure 1.

### 3.2. Molecular Classification of Endometrial Cancer

The choice of therapy is strictly dependent on the molecular subtype of endometrial cancer. Most commonly, the molecular subtype is assessed by the molecular testing of tumor tissue and immunohistochemistry. Next-generation sequencing (NGS) is also useful in the diagnosis of the molecular subtype, as it provides valuable information regarding microsatellite instability [14].

Specifically, there are four molecular types—endometrial cancer with mutations within the gene encoding epsilon polymerase (POLE Mutation POLEmut), with deficient mispaired bases (dMMR) and high microsatellite instability (MSI-H), with mutations in the TP53 gene and abnormal expression of p53 protein (p53abn), and endometrial cancer without a defined molecular profile (NSMP) [15]. Proficient mismatch repair (pMMR) refers to an intact DNA mismatch repair mechanism. It is not synonymous with a single molecular subtype but rather encompasses tumors classified as having no specific molecular profile (NSMP) and those with abnormal p53 expression (p53abn). pMMR tumors typically exhibit low tumor mutational burden (TMB), which results in lower immunogenicity compared to dMMR or MSI-H tumors. Consequently, pMMR tumors tend to respond poorly to immune checkpoint inhibitors such as pembrolizumab when used as monotherapy. However, clinical trials have demonstrated that the combination of pembrolizumab with lenvatinib—a multikinase inhibitor—can improve clinical outcomes in this patient population, likely through the modulation of the tumor microenvironment and enhancement in immune response [15].

The NSMP subtype is the most heterogeneous and largest group of endometrial cancer cases. This subtype of endometrial cancer is characterized by the absence of mismatch defects, pathogenic POLE variants, and p53 abnormalities. They often show a high expression level of progesterone and estrogen receptors [16]. It is considered a tumor with an intermediate prognosis. It is a subtype that is problematic in that it does not have a specific predictive biomarker [17]. Markers such as the overexpression of the L1 cell adhesion molecule (L1CAM), mutations in the β-catenin gene (CTNNB1), absence of estrogen and progesterone receptors, and amplification of the long arm of chromosome 1 (1q) are considered potentially useful for a more accurate differentiation and stratification of NSMP cancers [18].

The mismatch repair (MMR) system consists of seven key proteins responsible for detecting and correcting errors arising during DNA replication. These include the following: MutL homolog 1 (MLH-1), MutL homolog 3 (MLH-3), MutS homolog 2 (MSH-2), MutS homolog 3 (MSH-3), MutS homolog (MSH-6), and post-meiotic segregation increase 1 and 2 (PMS-1 and PMS-2) [19]. Of these, MLH-1, MSH-2, MSH-6, and PMS-2 are the most clinically relevant. These proteins form two basic complexes: MSH-2/MSH-6, which is responsible for the recognition of erroneous base pairs, and MLH-1/PMS-2, which initiates their repair process [20].

When one or more of these elements are not functioning properly or their expression is significantly reduced, MMR function is disrupted, resulting in the accumulation of mutations in the genetic material and leading to the phenomenon of microsatellite instability (MSI) [21].

Microsatellites are short, repetitive fragments of DNA located mainly in non-coding regions of the genome. Changes in their length are indicative of MSI, which is an indicator of increased cancer risk. MSI-H tumors are observed, among others, in colorectal cancer, especially in hereditary syndromes such as Lynch syndrome, also known as Hereditary Non-Polyposis Colorectal Cancer (HNPCC), but also in other cancers, including endometrial cancer [22]. In patients with HNPCC, particularly type II (involving extraintestinal tumors), the MSI-H/dMMR phenotype is associated with the presence of inherited, pathogenic mutations in the genes encoding the proteins of the MMR system [23]. This type of abnormality occurs in approximately 30% of endometrial cancers and is associated with a moderate prognosis—worse than in ultramutated tumors (e.g., with POLEmut) but more favorable than in aggressive subtypes with p53 mutations. A panel of five microsatellite markers is used to detect MSI, such as MLH1, MSH2, MSH6, and PMS2.

The p53abn subtype in turn refers to the p53 protein. It is a protein located in the cytoplasm and cell nucleus. It has an important function in the regulation of gene expression, apoptosis, and the cell cycle and controls and stimulates damaged DNA repair systems. p53 is a transcription factor that binds to DNA, thereby regulating gene diversity. In healthy cells, the levels of this protein are very low due to the control of its negative regulators Mouse Double Minute 2 Homolog (MDM2) and Murine Double Minute X (MDMX), which promote p53 degradation through ubiquitination [24]. TP53 is a tumor suppressor gene encoding the p53 protein, one of the most frequently mutated genes in cancer. The p53 subtype is associated with a poor prognosis and the highest mortality rate among endometrial cancer patients. It is usually detected by immunohistochemical staining for the abnormal expression of the p53 protein [25].

The last subtype is the POLEmut subtype, with a mutation in the gene encoding epsilon polymerase. Polymerase epsilon (Pol ε) is one of the key enzymes involved in DNA replication, alongside alpha and delta polymerases. It consists of four subunits, the largest of which, p261, is responsible for enzymatic activity, both catalytic and exonucleolytic [26]. The POLE gene specifically encodes the p261 subunit. During DNA copying, Pol ε is responsible not only for synthesizing a new strand but also for correcting errors through its ability to remove mismatched bases [27]. Mutations in the POLE gene result in the disruption of Pol ε repair function, leading to a significant increase in the number of mutations in tumor cells. In contrast to tumors with deficits in the MMR system, which are characterized by microsatellite instability and a moderately high number of mutations, tumors with POLE mutations retain microsatellite stability but have an ultra-high mutational burden [28]. Although the MMR system can partially compensate for Pol ε errors, it is not effective enough to prevent the accumulation of multiple genetic alterations. Importantly, mutations in POLE can co-occur with mutations in MMR genes, further exacerbating genomic instability [28]. The POLEmut tumor subtype is associated with a very favorable prognosis. This is thought to be due to an enhanced immune response against these tumors. POLE mutations lead to the up-regulation of the inflammatory response, thereby promoting the recruitment of cytotoxic T cells [16].

### 3.3. Pembrolizumab

The basis of immunotherapy is the regulation of B and T lymphocytes by the inhibition or activation of immune checkpoints. Antigen-specific and antigen-nonspecific signaling play a significant role in the regulation of immune system activation [29].

Antigen-specific signaling involves the recognition of tumor cells by T cells through the binding of the T-cell receptor (TCR) exposed on the surface of the lymphocytes to the major tissue compatibility complex (MHC) exposed on the surface of tumor cells [30]. In contrast, antigen-nonspecific signaling involves the synergistic action of the CD28 protein (on the surface of T lymphocytes) with the TCR. This is important in the context of regulating lymphocyte activity [31].

Therapies using monoclonal antibodies that target the interaction between the programmed cell death 1 (PD-1) receptor and its ligands, Programmed Death-Ligand 1 (PD-L1) and Programmed Death-Ligand 2 (PD-L2), have shown exceptional efficacy in treating and sometimes even completely curing cancers [32]. PD-1, a member of the CD28 family of receptors, is mainly present on the surface of activated T and B lymphocytes. Its interaction with natural ligands, PD-L1 or PD-L2, leads to the activation of signaling pathways responsible for inhibiting the T-cell immune response [33].

PD-L1 and PD-L2, which are found not only on healthy cells but also on the surface of many cancer cells, play an important role in enabling tumors to evade immune surveillance. PD-1 inhibitors, by competing with endogenous PD-L1, bind with high affinity to PD-1, leading to the restoration of T-cell activity and their ability to eliminate tumor cells [34].

**Figure 2:**
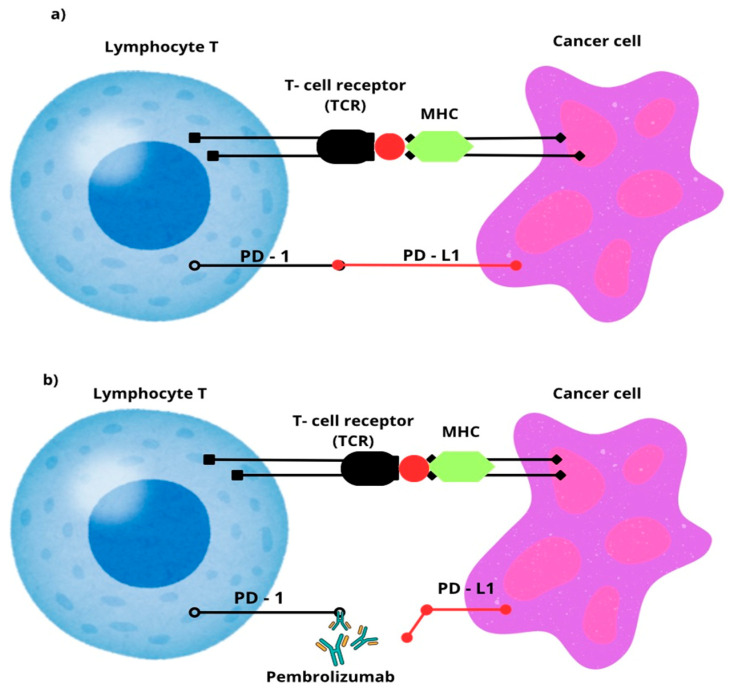
(**a**) depicts the interaction of the T-cell receptor (TCR) with the tissue compatibility system (MHC) of a tumor cell and shows how the interaction between PD-1 and PD-L1 inhibits T-cell activity. (**b**) shows the intervention of pembrolizumab—by attaching to the PD-1 receptor, it prevents the interaction of its ligand PD-L1, thus preventing the inhibition of T-cells [36].

Pembrolizumab is a humanized IgG4 monoclonal antibody that targets the PD-1 receptor on T lymphocytes. By blocking its interaction with the PD-L1 and PD-L2 ligands, pembrolizumab prevents immune suppression and restores T-cell activity against tumor cells [35]. Figure 2 illustrates the mechanism of action of pembrolizumab on tumor cells.

Pembrolizumab is a drug approved for melanoma, non-small cell lung cancer, and Hodgkin’s lymphoma, among others. The Food and Drug Administration (FDA) granted accelerated approval in 2017 for the anti-PD-1 antibody treatment of patients with MSI-H or dMMR tumors. In September 2019, it approved pembrolizumab in combination with lenvatinib for patients with pMMR or MSI-H tumors that had progressed after prior systemic therapy. In 2022, pembrolizumab received full approval as a single agent for endometrial cancer patients whose disease had progressed after earlier treatments [37].

National Comprehensive Cancer Network (NCCN) recommendations indicate that in patients with recurrent, metastatic, or high-risk endometrial cancer, the combination of carboplatin/paclitaxel with pembrolizumab, dostarlimab, or durvalumab is the preferred first-line treatment option. In the context of second-line treatment targeting biomarkers, the guidelines recommend pembrolizumab for patients with tumors showing MSI-H or dMMR, while for tumors without these features (i.e., pMMR/non-MSI-H), they recommend combination therapy with lenvatinib and pembrolizumab [38]. A summary of the recommendations for the inclusion of pembrolizumab according to the NCCN guidelines is presented in Table 1.

### 3.4. Research

The first double-blind RCT study included 816 patients aged 18 years and older with advanced endometrial cancer, metastatic or recurrent, of any histological type, excluding sarcomas [39]. The inclusion criteria were patients with confirmed stage III, IVA cancer with measurable disease or IVB, or recurrent cancer with measurable or non-measurable disease. Patients who had previously received adjuvant chemotherapy were eligible for inclusion, provided that the period without chemotherapy was 12 months or more, with prior hormone therapy or radiotherapy, and Eastern Cooperative Oncology Group (ECOG) performance status was 0, 1, or 2 (scale from 1 to 5, with higher scores indicating lower performance).

Patients were divided according to dMMR (n = 225) and pMMR (n = 591) disease. Each of these groups was then divided according to the drugs used: one received pembrolizumab, paclitaxel, and carboplatin, and the other received placebo, paclitaxel (intravenously, 175 mg per m^2^ of body surface area), and carboplatin (intravenously, 5 mg/mL/min for 30–60 min) for six cycles [39].

After this period, they received maintenance treatment with pembrolizumab or placebo (up to 14 cycles every 6 weeks). The initial dose of pembrolizumab and placebo administered intravenously was 200 mg during a 30 min infusion, and the maintenance dose was 400 mg. The observation period was 12 months. In the dMMR cohort, the 12-month PFS was 74% in the pembrolizumab group and 38% in the placebo group. The hazard ratio (HR) for progression or death was 0.3 (*p* < 0.001), representing a 70% reduction in the risk of progression or death. In contrast, the median PFS in the pMMR cohort was 13.1 months in the pembrolizumab group and 8.7 months in the placebo group (HR = 0.54; *p* < 0.001). Side effects in the pembrolizumab and placebo groups, respectively, were as follows: fatigue, 65.7% and 59%; sensory peripheral neuropathy, 58.2% and 58.7%; anemia, 55.8% and 53.2%; and thrombocytopenia, 31.4% and 23.7% [39]. Other side effects such as nausea, constipation, shortness of breath, and joint pain were slightly more prevalent in the pembrolizumab group. Overall, the incidence of side effects according to the authors’ calculations was 89.4% in the pembrolizumab group and 88.4% in the placebo group [39].

Another double-blind RCT was conducted on 1095 patients (median age 62 years) with newly diagnosed FIGO stage I/II endometrial cancer with non-endometrial or endometrial histology with TP53 abnormality or stage III or IVA with any histology [40]. The median follow-up was 24.6 months. The aim of this study was to test the efficacy of adding pembrolizumab to adjuvant chemotherapy (carboplatin and paclitaxel) on disease-free survival (DFS) and OS. Patients were divided into two groups receiving pembrolizumab (n = 545) and placebo (n = 550), according to pMMR and dMMR, with 74% and 26% of patients having this type of cancer, respectively, and allocation to groups was random. Pembrolizumab and placebo were administered at a dose of 200 mg every 3 weeks for six cycles, followed by 400 mg every 6 weeks for six cycles [40].

The results showed that the 2-year DFS was 75% in the pembrolizumab group and 76% in the placebo group. Side effects were higher in the pembrolizumab group (71%) than in the placebo group (63%). Based on these data, it was found that pembrolizumab did not provide the expected benefits in the entire population. Only patients with dMMR appeared to benefit from immunotherapy [40].

This study was a pre-specified subgroup analysis presented in a separate publication and focused only on patients with dMMR. They were divided into two subgroups: carboplatin and paclitaxel + pembrolizumab (n = 141) and carboplatin and paclitaxel + placebo (n = 140) [41]. The doses and regimen were the same as in the previous study. The 2-year DFS in the pembrolizumab group was 92.4% and in the placebo group 80.2%. The number of recurrences was 8 (pembrolizumab) and 25 (placebo), respectively. However, side effects were more frequent in the pembrolizumab group: anemia in 76 patients (70 in the placebo group), diarrhea in 67 patients (51 in the placebo group), and joint pain in 45 patients (43 in the placebo group). Overall, side effects occurred in 78.6% of patients treated with pembrolizumab (110 patients) and 66.4% in the placebo group (93 patients) [41].

Another double-blind RCT was conducted on a cohort of 827 patients, including 697 with pMMR and 130 with dMMR [42]. Inclusion conditions were age 18 years and older, with confirmed advanced, recurrent endometrial cancer of any histological type (excluding sarcomas and angiosarcomas) or metastasis, and 0 or 1 on the ECOG scale. Previously taken hormone therapy was not relevant. Patients were randomly allocated to groups. Group I comprised 411 patients who received pembrolizumab with lenvatinib. Group II received chemotherapy, including doxorubicin 60 mg per square meter of body surface area, intravenously every three weeks, or paclitaxel 80 mg per square meter, intravenously every week—three weeks on the drug and one week off. In the pMMR study group, PFS averaged 6.6 months in the pembrolizumab group and 3.8 months in the chemotherapy-treated group. In the general population, PFS was 7.2 months in the pembrolizumab group and 3.8 months in the chemotherapy group. OS was also longer in the pembrolizumab + lenvatinib group at 18.3 months and in the chemotherapy group at 11.4 months, with a pMMR population survival of 17.4 months (pembrolizumab + lenvatinib) and 12 months (chemotherapy), respectively. Furthermore, a decrease in tumor volume was observed in the pembrolizumab group [42].

Side effects were generally comparable, with 99.8 per cent of subjects experiencing any adverse effects in the pembrolizumab and lenvatinib groups and 99.5 per cent in the chemotherapy group, with hypothyroidism, hypertension, or diarrhea predominating in the former group [42].

In another RCT, 842 patients, with a median age of 63.5 years, with stage III–IV or recurrent endometrial cancer, were randomly allocated to groups: I—pembrolizumab and lenvatinib (n = 420, including 320 pMMR)—and II—chemotherapy (n = 422, including 322 pMMR) [43]. Patients in group I received pembrolizumab 200 mg once every three weeks and lenvatinib 20 mg once daily, and those in the chemotherapy group received paclitaxel 175 mg per 1 m^2^ body with carboplatin 6 mg/mL/min once every three weeks. This study was conducted from May 2019 to March 2025. The median time was a mean of 34.4 months. In the pMMR population, 53 patients (17%) out of 320 treated with lenvatinib and pembrolizumab and 51 patients (16%) out of 322 in the chemotherapy group received prior neoadjuvant or adjuvant chemotherapy. In the overall study population, 63 patients (15%) in the lenvatinib and pembrolizumab group and 58 patients (14%) in the chemotherapy group, respectively, had previously received neoadjuvant or adjuvant chemotherapy [43]. Of all patients, 442 (52%) died. The median OS was 37.7 months in the lenvatinib and pembrolizumab group and 32.1 months in the chemotherapy group. OS was similar between groups in most patient subgroups. The exception was patients who had previously received neoadjuvant or adjuvant chemotherapy—in these patients, the benefit of lenvatinib and pembrolizumab treatment was more pronounced in both the pMMR and overall population, as well as in the dMMR subgroup. A total of 76% of patients in group I reported treatment side effects and 66% of patients in group II [43].

A subsequent subgroup analysis of the Japanese population from the KEYNOTE-775 trial included patients over 18 years of age with confirmed recurrent, advanced, or metastatic endometrial cancer (patients with sarcomas and sarcomas were not classified) and ECOG grade 0 or 1 [44]. Patients were not disqualified if they received one additional line of chemotherapy or if it was a neoadjuvant or adjuvant treatment. Patients who had previously received any immunotherapy for PD-L1 or PD-L2 were excluded. A total of 104 patients were randomly allocated to two groups: I—oral lenvatinib 20 mg daily and intravenous pembrolizumab 200 mg every 3 weeks (n = 52, including 44 pMMRs and 8 dMMRs)—and II—chemotherapy, including intravenous doxorubicin 60 mg/m^2^ or paclitaxel 80 mg/m^2^ (n = 52, including 47 pMMRs and 5 dMRs), 3 weeks of treatment and 1 week off. The median follow-up time was 11.8 months. In the pMMR patients treated with pembrolizumab and lenvatinib, PFS was 5.6 months (HR = 1.04), the same as in the chemotherapy group. In all patients, the median PFS was 7.2 months and 5.4 months (HR = 0.81), respectively. In patients with dMMR, the median PFS was not achieved in either group (HR = 0.17) [44]. The median OS in patients in group I was 16.7 months and 12.2 months in group II (HR = 0.74). Patients with dMMR disease in group I achieved a median OS of 11.3 months in group I and 8 months in group II. Regarding side effects, they occurred in 90.4 per cent of patients in the pembrolizumab and lenvatinib groups and 84.4 per cent in the chemotherapy group. The most common adverse events were hypertension (78%), hypothyroidism (75%), and proteinuria (63.5%) in the pembrolizumab group and neutropenia (66.7%), nausea (60.8%), and anemia (47.1%) in the control group. All side effects from the treatments in the mentioned works are summarized in Table 2.

## 4. Discussion

Standard treatment for endometrial cancer relies primarily on radical surgery with adjuvant therapy, and the prognosis for advanced cancer is poor. The steady annual increase in morbidity and mortality from this cancer underscores the urgent need for improved treatment methods, making the use of new strategies that prolong survival, such as immunotherapy, crucial.

Regarding immunotherapy as a novel therapeutic approach in this setting, PD-1/PD-L1 inhibitors have begun to play a bigger role, which act by blocking the PD-1 checkpoint and allowing T cells to remain active. The most important aspect of this therapy is blocking the PD-1 checkpoint, which allows T cells to remain active and fight the tumor. Pembrolizumab is a key immune checkpoint inhibitor used in the treatment of endometrial cancer. It has been approved by the FDA for the treatment of lymphomas, kidney cancer, and bladder cancer [45].

In recent years, the introduction of this therapy has significantly improved the prognosis for patients with recurrent or advanced endometrial cancer. Initially, immunotherapy was used primarily as a second-line treatment and in cases of advanced or metastatic endometrial cancer that had not responded to prior treatment.

In March 2022, following an analysis of the KEYNOTE-158 study, the FDA approved pembrolizumab as monotherapy for patients with MSI-H or dMMR endometrial cancer who had disease progression after prior systemic therapy and were not candidates for definitive therapy [46,47]. Earlier, in September 2019, based on the KEYNOTE-146 study, the FDA granted accelerated approval for the combination of pembrolizumab with lenvatinib for patients with advanced endometrial cancer without MSI-H or dMMR who had disease progression after prior systemic therapy and were not candidates for definitive surgery or radiotherapy [42,48]. In June 2023, based on the results of the GY018 study, the indications for pembrolizumab were expanded to include first-line treatment in combination with chemotherapy and then as maintenance monotherapy in patients with advanced or recurrent endometrial cancer [49]. According to Eskander et al., the addition of pembrolizumab to standard therapy and subsequent pembrolizumab maintenance therapy resulted in a 70% lower risk of disease progression or death in patients with dMMR disease and a 46% lower risk in patients in the pMMR cohort compared with placebo. This suggests that the inclusion of immunotherapy in first-line treatment for patients with advanced or recurrent endometrial cancer improves outcomes, regardless of MMR status [39].

In the Makker study, patients experienced statistically less benefit in the pMMR group (74% of patients) than in the dMMR group, in which pembrolizumab appears to be more effective. After further study, the number of relapses significantly favored pembrolizumab [42].

Prognosis and improvement in the median OS and PFS varied depending on MMR status; therefore, molecular subtype should be carefully defined before enrollment or treatment initiation. dMMR tumors are characterized by a high mutational burden, leading to the development of numerous neoantigens that may increase tumor immunogenicity, making them more susceptible to immune attack. Furthermore, dMMR tumors often demonstrate increased PD-L1 expression and T-cell infiltration, making them more susceptible to checkpoint inhibitor therapy. The NCCN has also provided its recommendations for the use of pembrolizumab by molecular subtype, as defined in Table 1.

Median OS is a key patient outcome, so we based our analysis on it. The median OS in patients treated with pembrolizumab ranged from more than 18 months in the group with very advanced disease to more than 37 months. This median OS was not reached in many studies, such as ENGOT-en11/GOG-3053/KEYNOTE-B21 [40]. This indicates that patients treated with pembrolizumab were alive at the time of data analysis, suggesting a potentially long-lasting treatment effect and high efficacy. For example, a study included 79 patients with previously treated, unresectable, or metastatic endometrial cancer aged 42–86 years, ECOG 0 or 1. Of these, 48% had received one prior line of therapy, 52% had received two or more lines of therapy, and 68% had received prior radiotherapy. The median PFS was 13.1 months, with 51% of patients remaining relapse-free at 1 year. The median OS was not reached [50]. The median follow-up time in this study was 42.6 months, which suggests that the treatment effect is relatively good and efficient.

However, it is important to note that the omission of median OS is a significant detail. OS could not be estimated in the KEYNOTE-B21 study because too few patients died. However, this study enrolled patients after surgery, without prior radiotherapy or systemic therapy. This is important because the good outcomes of patients treated with pembrolizumab may not be due to the drug’s effect per se but rather to the completeness of the resection.

In the KEYNOTE-158 study, pembrolizumab was used in patients with dMMR disease who had not responded to prior therapy and had not undergone surgery. This population included patients with more advanced disease. In this case, the lack of median OS may be used to assess the true long-term effectiveness of immunotherapy.

It is important to note that all studies enrolled patients with good performance status—ECOG 0–1, sometimes 2. Translating this into clinical practice is difficult because the actual population of patients with advanced endometrial cancer often presents differently—they have multiple comorbidities and poorer performance status, which is often a criterion for excluding them from these medications. Furthermore, patient follow-up was limited, making it difficult to assess the actual effectiveness and comparability of the therapy over a long period of time.

Moreover, the effectiveness of therapy should not be assessed solely on the basis of PFS or OS, as patients’ quality of life is also important. There are no analyses providing information on the impact of immunotherapy on patients’ daily lives and functioning, and side effects such as hypertension or hypothyroidism may have impaired their quality of life. Furthermore, not all studies specifically analyzed molecular subtype. This lack of such analysis may lead to a biased assessment of treatment effectiveness. Comparing the aforementioned studies can also be problematic, as not all focused on the same endpoints, which may limit the reliability of the studies.

Moreover, all studies cited in this review were designed before 2023 and therefore compared pembrolizumab with conventional chemotherapy. More immunotherapies, such as dostarlimab, have now been introduced for the treatment of endometrial cancer. To contextualize the results of these two agents, it is important to analyze the results of individual studies. In the KEYNOTE-158 study, which enrolled patients over 18 years of age with advanced/metastatic endometrial cancer with progression or intolerance to standard therapies, pembrolizumab (once every 3 weeks for 35 cycles) demonstrated an ORR of 57% in patients with advanced dMMR/MSI-H endometrial cancer [46]. Separately, the GARNET study evaluating dostarlimab (500 mg every 3 weeks for 4 cycles) in a similar patient population showed an ORR of 45.5% [50]. The median PFS was 13.1 months for pembrolizumab and 6.0 months for dostarlimab. In patients with dMMR/MSI-H, both pembrolizumab and dostarlimab as monotherapy are effective and well tolerated. However, in patients with pMMR, pembrolizumab in combination with chemotherapy may yield better results compared to dostarlimab and chemotherapy.

However, these studies are not compared well methodologically—KEYNOTE-158 enrolled previously treated patients, while GARNET enrolled patients after disease progression, also with advanced disease, after two or more lines of treatment, and the population was smaller and more molecularly structured [46,51].

In first-line treatment, the GY018 and RUBY studies evaluated pembrolizumab and dostarlimab in combination with chemotherapy, respectively. In the pMMR/microsatellite stable (MSS) endometrial cancer population in the GY018 study, pembrolizumab in combination with chemotherapy resulted in a PFS of 74% (HR = 0.3) in dMMR and 13.1 months (HR = 0.54) in pMMR [39]. In the RUBY study, for which patients with primarily advanced endometrial cancer (stage III or IV) or with first recurrent cancer were qualified, dostarlimab (500 mg) in combination with chemotherapy (carboplatin 5 mg/mL/min and paclitaxel 175 mg/m^2^) provided a two-year OS of 67.7% (HR = 0.73) and PFS in 28.4% (HR = 0.76) of patients at 24 months in pMMR and 61.4% (HR = 0.28) PFS and 83.3% (HR = 0.30) OS in dMMR [52]. However, the effectiveness of comparing these studies is minimal, as they are not head-to-head studies, and the inclusion criteria for patients differed—although the study results seem to favor pembrolizumab. However, it is difficult to find studies that are methodologically ideal for comparison, as this is a relatively new topic.

In this context, it is worth highlighting the latest research in this field. One of the most important study is AtTEnd—a randomized, double-blind, phase III study involving 549 patients over 18 years of age, with advanced or metastatic endometrial cancer or carcinosarcoma, ECOG 0–2, and have not previously received systemic chemotherapy for relapse. The efficacy of atezolizumab (1200 mg) added to chemotherapy (carboplatin at area under the curve of 5 or 6 and paclitaxel 175 mg/m^2)^ and as maintenance therapy was assessed. In the dMMR group, the median PFS was not reached in the atezolizumab arm, while in the placebo group, it was 6.9 months (HR = 0.36); similarly, the median OS was not reached in the study group and was 25.7 months (HR = 0.41) in the control group. No significant differences were demonstrated in the pMMR group [53]. The incidence of side effects was similar. However, the failure to reach the median OS means that the data are still immature and may change in the long term. Looking at both dostarlimab and atezolizumab, although these are still not head-to-head studies, it can be concluded that dostarlimab demonstrate high efficacy in the treatment of endometrial cancer. Atezolizumab, on the other hand, requires further studies to fully assess its effectiveness. Moreover, dostarlimab is FDA-approved for the treatment of endometrial cancer, unlike atezolizumab, making dostarlimab a more reliable treatment choice [54].

Research in this area is increasingly advanced, and therapeutic options in the care of cancer patients are expanding. These studies once again confirm the crucial importance of molecular subtype.

Nivolumab (anti-PD-1), approved by the FDA; atezolizumab and durvalumab (PD-L1 inhibitors), which are currently in clinical trials; and cabozantinib, a tyrosine kinase inhibitor, are also used in the treatment of endometrial cancer. For example, a randomized phase II trial evaluated the efficacy of cabozantinib in combination with nivolumab in patients with recurrent endometrial cancer, where the combination therapy demonstrated superiority over monotherapy, even in a population with prior immunotherapy [55]. Interestingly, the phase II POD1UM-204 study is ongoing in patients with advanced or metastatic endometrial cancer after progression following platinum chemotherapy. Participants were assigned to cohorts based on molecular profiles and received retifanlimab (PD-1 inhibitor) as monotherapy or in combination with epacadostat—inhibitor of indoleamine 2,3-dioxygenase (IDO-1); pemigatinib—inhibitor of fibroblast growth factor receptors (FGFRs); or Lymphocyte-Activation Gene 3 (LAG-3/TIM-3) inhibitors. The study began in January 2021 and is scheduled to conclude in July 2026 [56]. Based on the results of these studies, clinical knowledge may significantly expand to include previously questionable aspects and new discoveries.

Another important aspect is the occurrence of side effects of therapy. In terms of safety, immunotherapy was associated with frequent but usually predictable side effects. The most frequently observed in studies involving pembrolizumab and its combinations included hypothyroidism, hypertension, proteinuria, and gastrointestinal symptoms such as diarrhea and nausea. In comparative studies with chemotherapy, the incidence of side effects was similar. In the immunotherapy group, side effects related to the development of autoimmunity predominated. These were likely not very persistent, as few patients discontinued treatment because of side effects. Furthermore, no study reports indicate whether these side effects significantly affected patient functioning or whether, and if so, with which medications, they were treated. Only in the study by Makker based on the QLQ-C30 questionnaire was it found that the quality of life of patients in both groups did not differ significantly. Future reports should definitely consider the subjective well-being of patients.

There is also increasing evidence of a correlation between the occurrence of immune-related adverse events (irAEs) and the efficacy of immune point inhibitors in the treatment of various cancers. In a meta-analysis encompassing 30 studies (4971 patients with lung cancer, melanoma, and renal cancer), the authors reported that patients who experienced irAEs lived longer and had slower disease progression than those who did not (OS HR = 0.54; PFS HR = 0.52; *p* < 0.001). The strongest beneficial effect was demonstrated for endocrine irAEs such as thyroiditis or hypophysitis. Furthermore, irAEs above grade 3 did not provide additional benefit and required additional immunosuppressive treatment, which could have impaired the efficacy of the therapy. Particular benefits were confirmed mainly for PD-1 inhibitors [57]. A particularly important example is the case report of a 64-year-old patient with recurrent MSI-H/dMMR endometrial cancer treated with pembrolizumab. After 20 cycles of pembrolizumab, severe colitis with hypokalemia recurred; treatment was discontinued. At the time of publication, the patient had been in complete remission for 22 months, despite discontinuing pembrolizumab therapy [58]. Another case report describes severe toxicity after a single dose of pembrolizumab but with a durable and strong antitumor response in a patient with Lynch syndrome. No recurrence of endometrial cancer was observed 20 months after drug administration. Such complications are rare and can be life-threatening for patients, but in some, they may paradoxically indicate effective immune activation [59].

Interestingly, the patients in the manuscripts cited in this review rarely exhibited typical endocrine side effects. This does not mean that pembrolizumab’s efficacy is lower in these patients, but considering that their average OS did not exceed 19 months (compared to the two patients mentioned above) or that the data were not mature enough, this fact supports this thesis. The authors themselves do not mention this correlation. The topic of correlation between the occurrence of irAEs and the effectiveness of checkpoint inhibitors remains controversial, and the mechanism of this reaction is not well understood. The most frequently cited theory is antigenic mimicry, where activated T lymphocytes attack both tumor cells and healthy tissue. However, much research is still needed to draw clear conclusions, particularly in endometrial cancer.

In summary, PFS and OS benefits were observed in patients with advanced, recurrent, or metastatic endometrial cancer treated in both first-line and second-line settings, with particularly favorable results observed in the dMMR/MSI-H group. However, it was not reported whether any symptomatic treatment was used in response to side effects and even if so, whether patients felt better after symptomatic treatment or whether adverse events were unaddressed.

This systematic review also has several limitations. Although RCTs are the gold standard for assessing treatment effectiveness because they minimize the risk of bias through random assignment, they have many limitations. One key issue is that the subjects included in such studies are carefully selected, meaning that they often differ from those encountered in everyday clinical practice. This review included patients with ECOG status 0–2 and excluded patients with sarcomas and sarcomatoid tumors. Furthermore, the care provided to participants in such a study may differ from the actual, everyday standards of care outside of the study setting. Both patient characteristics and human factors, such as the involvement of healthcare professionals, may differ significantly from those in the real healthcare system. Given the above considerations, translating the results of RCTs, and therefore this systematic review, to the general population may be difficult. Another important aspect is the difference in interventions—not all treatment regimens were similar, with the only common aspect being the use of pembrolizumab. Furthermore, the studies included in this review analyzed molecular subtypes in a limited or imprecise manner. The fact that the cited studies only cover the last 5 years (2020–2025) limits the possibility of including broader long-term data, but no further studies on this topic are currently available. The included studies also have different follow-up periods and different numbers of cycles, which may negatively impact the comparability of the results. Table 3 summarizes the studies included in this review.

## 5. Conclusions

Pembrolizumab is the most effective in patients with endometrial cancer characterized by dMMR and MSI-H. In this group of patients, clear benefits of immunotherapy were observed, including a significantly higher percentage of patients remaining disease-free after two years of treatment and fewer recurrences compared to standard therapy.

In patients with pMMR, the efficacy of pembrolizumab as monotherapy was limited. However, in combination with lenvatinib, a clear prolongation of overall survival and progression-free survival was observed compared to conventional chemotherapy. Nevertheless, not all studies showed a clear improvement in this group, suggesting a wide variety of responses to treatment and highlighting the need for careful molecular qualification before starting therapy. Immunotherapy with pembrolizumab, especially in combination regimens, contributed to a significant prolongation of overall survival in the entire patient population analyzed. In some cases, median OS was not reached, which may indicate the long-term benefits of treatment.

In terms of safety, side effects occurred frequently, but their incidence was comparable to that observed in groups receiving chemotherapy. The most commonly reported side effects in immunotherapy were hypothyroidism, hypertension, and proteinuria, but they did not lead to the discontinuation of treatment.

In summary, pembrolizumab is an effective therapeutic option for the treatment of advanced, recurrent, or metastatic endometrial cancer. The results clearly confirm the importance of molecular tumor assessment before making treatment decisions and highlight the growing role of immunotherapy in the treatment of endometrial cancer. It should be remembered that the limitations given by the authors and the variability in treatment regimens are the basis for further research on the efficacy of pembrolizumab in endometrial cancer.

## Figures and Tables

**Table 1:** Indications for pembrolizumab according to biomarkers. TMB-H—tumor mutational burden-high; dMMR—deficient mismatch repair; MSI-H—microsatellite instability-high; pMMR—proficient mismatch repair; uLMS—uterine leiomyosarcoma [38].

Indication	Use of Pembrolizumab
Cancers with dMMR/MSI-H	Monotherapy as the preferred option
Cancers with TMB-H	Monotherapy (as the second line)
Cancers with pMMR	Combination with lenvatinib
Uterine LMS with TMB-H	Second-line treatment

**Table 2:** Summary of selected treatment side effects from all included studies [39,40,41,42,43,44].

Author and Type of Intervention	Side Effects	Intervention: Frequency [%]	Intervention: Severity ≤ 3 Grade [%]	Control Group: Frequency [%]	Control Group: Severity ≥ 3 Grade [%]
Slomovitz BM. et al. Pembrolizumab + chemotherapy	Anemia	54.3%	0%	50%	9.3%
	Diarrhea	47.9%	2.9%	36.4%	3.6%
	Neutropenia	23.6%	17.1%	23.6%	14.3%
	Hypothyroidism	22.1%	0.7%	3.6%	0%
	Hyperthyroidism	11.4%	0%	3.6%	0%
	Thyroiditis	3.6%	0%	0.7%	0%
	Arthritis	<1%	0%	0%	0%
Marth C. et al. Pembrolizumab + lenvatinib	Hypertension	63%	43%	3%	1%
	Hypothyroidism	59%	1%	<1%	0%
	Diarrhea	42%	8%	16%	<1%
	Proteinuria	31%	7%	1%	<1%
	Neutropenia	4%	1%	30%	23%
Van Gorp T. et al. Pembrolizumab + adjuvant chemotherapy	Anemia	52%	11%	52%	9%
	Diarrhea	40%	2%	38%	3%
	Decreased neutrophil count	35%	23%	32%	22%
	Hypothyroidism	23%	<1%	4%	0%
	Hyperthyroidism	12%	0%	3%	0%
	Thyroiditis	2%	0%	<1%	0%
	Myositis	<1%	0%	<1%	0%
	Arthritis	<1%	0%	0%	0%
Eskander RN. et al. Pembrolizumab added to chemotherapy	Fatigue	65.7%	<1%	59%	<1%
	Sensory peripheral neuropathy	58.2%	<1%	58.7%	<1%
	Anemia	55.8%	15.3%	53.2%	<1%
	Thrombocytopenia	31.4%	<1%	23.7%	<1%
	Hypothyroidism	13.2%	0%	<1%	0%
	Hyperthyroidism	6.7%	0%	2.8%	0%
	Myositis	<1%	0%	0%	0%
Makker V. et al. Pembrolizumab + lenvatinib	Hypertension	64%	37.9%	5.2%	2.3%
	Hypothyroidism	57.4%	1.2%	0.8%	0%
	Diarrhea	54.2%	7.6%	20.1%	2.1%
	Proteinuria	28.8%	5.4%	2.8%	0.3%
	Anemia	26.1%	6.2%	48.7%	14.7%
Yonemori K. et al. Pembrolizumab + lenvatinib	Hypertension	78.8%	30.8%	0%	0%
	Hypothyroidism	75%	0%	0%	0%
	Proteinuria	63.5%	17.3%	7.8%	2%
	Platelet count decreased	48.1%	11.5%	13.7%	2%
	Anemia	42.3%	13.5%	47.1%	19.6%

**Table 3:** A table summarizing the studies included in this review [39,40,41,42,43,44].

Author and Year	Number of Patients	Intervention	Type of Examination	Results
Slomovitz BM. et al., 2025	281	Pembrolizumab + chemotherapy vs. placebo + chemotherapy	RCT, double-blinded	**DFS** 2 years: 92.4% vs. 80.2%; fewer relapses: 8 vs. 25 **OS:** the results were only 3.6% mature, so it was not mentioned; **PFS:** not reported
Marth C. et al., 2025	842	Pembrolizumab + lenvatinib vs. chemotherapy	RCT, double-blinded	**DFS** 2 years: 92.4% vs. 80.2%; fewer relapses: 8 vs. 25 **OS:** the results were only 3.6% mature, so it was not mentioned; **PFS:** not reported
Van Gorp T. et al., 2024	1095	Pembrolizumab + adjuvant chemotherapy vs. placebo + chemotherapy	RCT, double-blinded	**DFS** 2 years: 92.4% vs. 80.2%; fewer relapses: 8 vs. 25 **OS:** the results were only 3.6% mature, so it was not mentioned; **PFS:** not reported
Eskander RN. et al., 2023	816	Pembrolizumab added to chemotherapy (carboplatin + paclitaxel) vs. placebo with chemotherapy	RCT, double-blinded	**DFS** 2 years: not reported **OS:** not mentioned **PFS:** dMMR: 12 months 74% vs. 38% (HR = 0.3); pMMR: 13.1 vs. 8.7 months (HR = 0.54)
Makker V. et al., 2022	827	Pembrolizumab + lenvatinib vs. chemotherapy	RCT, double-blinded	**DFS** 2 years: not reported **OS:** pMMR: 17.4 vs. 12 months, overall: 18.3 vs. 11.4 months, (HR = 0.62) **PFS:** pMMR: 6.6 vs. 3.8 months, (HR = 0.6) overall 7.2 vs. 3.8 months;
Yonemori K. et al., 2022	104	Pembrolizumab + lenvatinib vs. chemotherapy	RCT, double-blinded	**DFS** 2 years: not reported **OS:** pMMR 16.7 vs. 12.2 (HR = 0.74) dMMR not reached vs. 8 months (HR = 0.11) **PFS:** pMMR 5.6 vs. 5.6 months (HR = 1.04) dMMR not reached vs. 3.7 months (HR = 0.17) overall: 7.2 vs. 5.4 months, (HR = 0.81)

## Data Availability

Data sharing not applicable—no new data were created or analyzed in this study.

## Abbreviations

The following abbreviations are used in this manuscript:

ASMR	Age-Standardized Mortality Rate
CBR	Clinical Benefit Rate
CTNNB1	β-Catenin Gene
DFS	Disease-Free Survival
dMMR	Deficient Mismatch Repair
ECOG	Eastern Cooperative Oncology Group
FDA	Food and Drug Administration
FGFR	Fibroblast Growth Factor Receptor
FIGO	International Federation of Gynecology and Obstetrics
HNPCC	Hereditary Non-Polyposis Colorectal Cancer
HR	Hazard Ratio
IDO1	Inhibitor of Indoleamine 2,3-Dioxygenase
IgG G4	Immunoglobulin G4
irAEs	Immune-related adverse events
L1CAM	L1 Cell Adhesion Molecule
LAG-3	Lymphocyte-Activation Gene 3
MDM2	Mouse Double Minute 2 Homolog
MDMX	Murine Double Minute X
MHC	Major Tissue Compatibility Complex
MMR	Mismatch Repair
MLH-1	MutL homolog 1
MLH-3	MutL homolog 3
MSH-2	MutS homolog 2
MSH-6	MutS homolog 6
MSI-H	Microsatellite Instability-High
MSS	Microsatellite Stable
NCCN	National Comprehensive Cancer Network
NGS	Next-Generation Sequencing
NSMP	No Specific Molecular Profile
ORR	Overall Response Rate
OS	Overall Survival
OSF	Open Science Framework
PD-1	Programmed Cell Death Protein 1
PD-L1	Programmed Death-Ligand 1
PD-L2	Programmed Death-Ligand 2
PFS	Progression-Free Survival
pMMR	Proficient Mismatch Repair
PMS-1	Post-Meiotic Segregation Increase 1
PMS-2	Post-Meiotic Segregation Increase 2
POLEmut	POLE Mutation
Pol ε	Polymerase Epsilon
p53abn	p53 abnormal
RCTs	Randomized Controlled Trials
TCR	T-cell Receptor
TMB	Tumor Mutational Burden
TMB-H	Tumor Mutational Burden-High
uLMS	Uterine Leiomyosarcoma
